# Exploring the role of digital media dependency on the relationship between personal involvement and flashbulb memory during the pandemic: Empirical evidence from Mainland China

**DOI:** 10.3389/fpsyg.2022.985287

**Published:** 2022-11-11

**Authors:** Xiaoyue Ma, Jing Wan

**Affiliations:** School of Journalism and New Media, Xi’an Jiaotong University, Xi'an, China

**Keywords:** flashbulb memory, digital media dependency, personal involvement, empathy, pandemic

## Abstract

Flashbulb memory (FBM) is viewed as a special type of autobiographical memory due to its richness of individuals’ self-related details when hearing the news and the long duration. It also helps shape people’s impression of public events to some extent. Given that personal involvement is one of the important antecedent variables of FBM, this study proposed to investigate it from spatiotemporal involvement (spatiotemporal distance) and empathic involvement (empathy level) to explore the impact of personal involvement on the formation of FBM during the Covid-19 pandemic. In particular, digital media dependency was considered in the influence of involvement on the FBM since it is a crucial information source for individuals and a path to spread information about their lives and work during the pandemic. In this study, a total of 546 valid questionnaires (from May 1, 2022, to May 7, 2022) and 349 valid questionnaires (from May 10, 2022, to May 17, 2022) were collected through a two-stage online survey in Shanghai, China towards the epidemic wave at the end of March 2022. The mediating mode of digital media dependency was also examined on personal involvement in FBM performance, which consists of FBM specificity, confidence, and consistency. Results showed that empathic involvement had a significant negative influence on FBM specificity, namely the higher the empathy level was, the worse the FBM specificity would be, in which digital media dependency played a suppressing effect. Individuals’ spatiotemporal involvement was proved to have a significant positive influence on FBM specificity and consistency. It was one of the first to investigate the FBM formation process around “small peak” events in the context of the ongoing pandemic. Innovatively, empathy was adopted as the index of memory arousal for empathic involvement, and digital media dependency was considered an important mediator variable in the memory study. The research results have practical significance for promoting the process of epidemic recovery integrated with digital media and can provide a social reference for the shaping process of disaster memory from the perspective of digital information and emotional transmission.

## Introduction

In this “extraordinary moment in history” (Historic England, 2020), individuals are documenting Covid-19 through various means such as online commemorative events and blogging using digital media, to try to construct a characteristic memory of the pandemic (Adams and Kopelman, 2021). Memory is regarded as a valuable tool for providing guidance and consolation by evoking associations from the present to the past (Simko, 2015; Liashenko et al., 2020), especially in times of crisis and uncertainty (McKinnon, 2019). Although many countries such as Denmark have lifted almost all Covid curbs to stop referring to Covid-19 as a “society-critical disease” (Financial Times, 2022), under the “Dynamic Zero Covid” policy of mainland China, the cycle of “outbreak-control - zero clearance” caused by imported cases can still bring about “small peak” epidemic events and aroused individuals’ attention. By now, as a “new normal” created by the pandemic (Norman, 2021), individuals have already become accustomed to living with Covid-19 (Kong et al., 2022), while intermittent outbreaks of “small peak” events can evoke people’s memories (cognitive, emotional, etc.) related to the pandemic which has last for 2 years more. During this period, information was widely disseminated through digital media, while individuals’ personal experiences and memories during the epidemic were “archived” online profit from the technological features of digital media (Liew et al., 2014), adding not only a new awareness of the pandemic but also shaping a new impression of the epidemic.

Covid-19 was unique in that it affected nearly everyone around the globe, as opposed to other public events which have a limited impact on a limited number of people. Events related to the Covid-19 pandemic are likely to leave their mark on history, transcending individual borders and being retained as part of the collective memory of nations and the world, with worldwide significance. As an event universally experienced by individuals around the world, the Covid-19 pandemic has created a unique circumstance to examine the factors of collective memory (Öner et al., 2022). Memories related to Covid-19 can serve to create a sense of self-continuity, guide present to future behavior, and provide social connections to others (Wolf and Nusser, 2021). The level of continuity of life before and after the pandemic is still uncertain, however. As society grapples with such a pressing issue, it is informative to examine the cognition and emotion of individuals to ease the post-pandemic life recovery process and to provide experience and reliability in similar future circumstances (Vanaken et al., 2021).

As a specific type of autobiographical memory, flashbulb memory (FBM) is often used for unexpected, traumatic, vivid, and important personal or national events (Er, 2003), which refers to individuals’ memory of details related to their surroundings at the time of being informed of the event or news (Berntsen, 2009). Most of the previous studies on FBM, on the one hand, were conducted on immediate events which are always happened in the past and had a definite beginning and end time mark, such as the 9/11 terrorist attacks (Curci and Luminet, 2006; Hirst et al., 2009; Kvavilashvili et al., 2010) or the death of a well-known person (Brown and Kulik, 1977; Day and Ross, 2014), earthquakes (Neisser et al., 1996), etc., while few studies have explored FBMs of long duration and ongoing events such as the pandemic. On the other hand, personal involvement in an event has been shown to be significant for flashbulb memories (Er, 2003), but “involvement” is not a unified concept and takes different forms across events (Neisser and Harsch, 1992). In some specific situations, the concept of “involvement” has been materialized into the importance/consequentiality of the event to individuals, that is, individuals’ degree of concern about the original event, which depends on factors such as ethnic groups (Brown and Kulik, 1977), social/national membership (Kvavilashvili et al., 2003; Curci and Luminet, 2006; Tinti et al., 2009), political stance (Raw et al., 2021), religious faith (Tinti et al., 2009; Curci et al., 2015) and geographic proximity (Pezdek, 2003). However, others have defined the concept with an emotional tinge attached to whether individuals have a direct experience of the event (Neisser et al., 1996; Er, 2003), which contributes to their different levels of psychological distance (Tekcan et al., 2003) and emotional arousal (Smith et al., 2003; Lanciano et al., 2010) and eventually affect individuals’ FBMs (Talarico and Rubin, 2007, 2018).

In addition to being a virus, Covid-19 represents a global crisis in human history (Gonçalves et al., 2020). People not only face the threat of susceptibility to the virus (Miller et al., 2021), but also face the concerns such as medical resources shortage (Nourkova and Alena, 2022), lockdown of loneliness, and isolation (Wolf and Nusser, 2021). It is therefore sensible to consider both the psychological and the physical dimensions of people’s involvement in events associated with Covid-19. In terms of the physical dimension, individuals’ spatial location (Granell et al., 2013) and the time span (McKinnon, 2019) were proved to have a significant impact on people’s memory of disaster events such as epidemics. It is found necessary to consider the spatiotemporal information when investigating the public reactions to Covid-19 (Feng and Zhou, 2022). While in terms of the psychological dimension, studies have linked autobiographical memory with empathy, such as using film to indicate that dispositional empathy facilitates the construction of memory (Harris et al., 2004) and one’s memory can in turn construct models to empathize with others’ inner world (Bluck et al., 2013). In disaster events, individuals’ ability to empathize affects their autobiographical memories, which include feelings of warmth, compassion, and sympathy for the victims (Franca et al., 2014). Based on the previous works on empathy during Covid-19 including its impact on prosocial behaviors (Jiang et al., 2021), vaccination intentions (Pfattheicher et al., 2022), and its traits (He et al., 2022), etc., this study is supposed to investigate whether empathy, as an ability to feel and imagine others’ conditions (Decety and Jackson, 2004), affects people’s memory of the events related to Covid-19.

In particular, in the age of digital media dominance (Poell, 2020), digital media are thought to play an important role in the propagation of epidemic events (Bhagya-Lakshmi and Rajaram, 2012). Since the early 2020s, individuals had demonstrated a dramatic increase in digital media use to meet the “social distance” requirement – they desperately need information online about where to go or what to do next during the pandemic (Burel et al., 2021). In addition, medical organizations and institutions tended to use digital media platforms to disseminate medical consensus and expert opinions related to the epidemic, and digital media had thus become a common channel for people to access information about the epidemic (Wong et al., 2021). On the one hand, digital media allows people to maintain a maximum normal life and to express their views and exchange views with others while maintaining social distance (Liu et al., 2022), while on the other hand, social isolation (in the case of lockdowns and quarantines) leads to an unconscious dependency on digital media use (Nabity-Grover et al., 2020) and whether this dependency has an impact on individuals’ memories of the epidemic has little been explored. As such, this study specifically considered the role of digital media in studying flashbulb memories.

Since FBM contains many details that are relevant to the individuals themselves and is full of personal uniqueness and individual characteristics (Brown and Kulik, 1977), this kind of memory further deepens the irreplaceability of the pandemic. In order to make a clearer depiction of individuals’ personalized memory during the epidemic and explore the antecedents of the formation, based on the enlightenment of previous studies on the heterogeneous form of “involvement” and empathy as influencing factors of memory, this study intends to explore the influence of personal involvement on FBM in terms of spatiotemporal involvement and empathic involvement in the context of intermittent “small peak” epidemic events in cities of mainland China where the fight against the epidemic is not seen to be over. Spatiotemporal involvement is determined by a person’s spatial distance and time span from the event, while empathic involvement is determined by the level of empathy. In particular, the role that digital media dependency plays in facilitating or hindering this influence path was also examined. This study aims to answer the following research questions (RQs): (RQ1) how do individuals’ personal involvement, including both the physical and the psychological aspects in Covid-19 events, affect FBM performance when the epidemic has not yet been declared over? (RQ2) does digital media dependency play a role in the impact of personal involvement on FBM performances and what role does it play?

This study follows the methodology of previous studies on FBM with questionnaires and self-reported indicators (Lanciano et al., 2013), and takes the research purpose into consideration to use a two-phase online questionnaire including the test phase and the retest phase to measure individuals’ FBM performance during the outbreak of Covid-19 in Shanghai, one of the metropolises in Mainland China, in late March 2022. A total of 546 valid questionnaires (From May 1, 2022, to May 7, 2022) and 349 valid questionnaires (from May 10, 2022, to May 17, 2022) were collected through a two-stage online questionnaire survey including the test phase and retest phase. The purpose was to measure individuals’ FBM performance of the outbreak “small peak” event, as well as their personal involvement in the event and their digital media dependency. In the test phase, respondents were asked to fill in the standard FBM questionnaire including six items related to the Shanghai outbreak, and to report their level of confidence in their responses in order to measure FBM confidence, while their location and the time span (the proxy of spatiotemporal involvement), empathy level (the proxy of empathic involvement) and digital media dependency were examined using the corresponding scales. In the retest phase, the same participants as in the test phase were invited to report six items including (i)the time when they heard the news, (ii) the source of information, (iii) their location, (iv) who they were with, (v) what they were doing beforehand and (vi) what they did after again. Then the responses in the two phases were compared to determine FBM consistency.

The innovations of this study are illustrated as follows. Firstly, different from previous studies on Covid-19 that were conceptual, macroscopic, and case-based, this study explored people’s memory of major epidemics from a more microscopic and personalized perspective, introducing the concept of flashbulb memory. Besides, FBM studies used to focus more on immediate events, while this study aimed at the intermittent “small peak” epidemic events in the context of the “new normal” epidemic to examine the FBM of continuous events. When a new “small peak” event occurred, relevant information would also emerge, which would shape new memories or change previous memories for individuals. This study emphasized the individuals’ personalized memory during the time when the event is still ongoing. Secondly, this study classified personal involvement into two aspects where spatiotemporal involvement is assessed by the spatial location and time span, and empathic involvement is measured by the level of empathy, which refined the connotation of personal involvement as an antecedent variable of FBM. During this peculiar period, individuals know about the occurrence and progress of the “small peak” pandemic event mostly from long-distance transmitted information. Moreover, this study innovatively transferred empathic involvement, as a concept in the clinical field, to memory research to investigate its prediction on FBM. This study described the formation of individuals’ long-distance cognition and memory of the event from both physical and psychological aspects, enriching the concept of involvement. Finally, this study originally introduced the variable of digital media dependency, considering the role of digital media in the formation of individual pandemic memory in the era of dominant information dissemination. For maintaining a normal life in the midst of a pandemic, digital media has become a widespread tool. Still, many people are not aware of the results of their dependence on digital media, especially in memory formation. This study innovatively considered the impact of digital media on individual memory formation and analyzed its suppressing effect.

The rest of the paper is organized as follows. The next section provides the theoretical development. In section 3, the hypotheses were proposed. The empirical research and data analysis were demonstrated in section 4. Section 5 displayed the results while the explanation and implications were discussed in section 6. Finally, we concluded and prospected the future work of this study.

## Literature review and theoretical background

### Disaster memory, Covid-19, and digital media

Disaster experiences could become part of the history of those affected and would have an important impact on the recovery process for individuals could gain experience from memories of catastrophic events to develop better coping strategies to handle future risks (Folke et al., 2005; Oliver-Smith et al., 2016). Based on the fact that disasters are largely a social construct (Kelman, 2020), the memories of people in the affected areas will also become a significant element of the collective memory and will develop a defining place within local identities (Mckinnon et al., 2016; McKinnon, 2019). And, direct experiences such as witnessing a disaster, and indirect experiences such as information from others, education, and media play a major role in memories recalling previous disasters (Wachinger et al., 2013). The mental state (Philippe et al., 2019) and emotional response (Wu, 2020) of individuals after disasters may change over time, depending on personal differences, and individuals may remember consequential events related to themselves for long periods of time (Schuman and Scott, 1989). Moreover, disaster experiences can affect individuals’ cognition of disaster, such as the emotion of fear or threat, the perception of protective behaviors, the trust in external prompts, and the remembrances or thinking about the event (Helton et al., 2011; Philippe and Houle, 2019; Wei et al., 2020). Particularly, memories of the disaster are found to maintain longer than related emotions (Knez et al., 2018). As a global disaster that is still ongoing, Covid-19 has assumed the form of a “traumatic memory in its making” (Demertzis and Eyerman, 2020). Although epidemics are widespread throughout the history of human existence, before the Covid-19 pandemic, there was little documentation of major epidemics in society through digital formation such as social media. People’s “epidemic memory” seems to exist only in the scientific realm relating to virology, epidemiology, and the development of vaccines (Vinitzky-Seroussi and Jalfim, 2021). Owing to the accessibility of technology which has enabled people to use digital media to document their daily lives at all times and places, Covid-19 is unfolding in and shaping a new memory boom that becomes detached from any kind of reality of the past. It is the memory from the present and also for the present (Hoskins and Halstead, 2021).

The role of digital media during the pandemic is to provide information about Covid-19 and health-related issues, to meet people’s needs for online work and entertainment, and to serve as a place to share emotions and record memories (Wong et al., 2021). Since the outbreak of the pandemic, the use of digital media has become increasingly important for health and crisis communication (Piper, 2020). A study found that more than 90% of the participants obtained Covid-19 information from the internet and they were keen to know more about Covid-19, including the Covid-19 transmission route, the medication, and vaccine availability, and effectiveness, travel advice, tailormade information for different populations, etc. (Wang et al., 2020). Since digital media such as Twitter and Facebook now are the principal sources of information for the public, they become fundamental drivers of people’s perceptions and opinions and thus of their behaviors (Gozzi et al., 2020). Meanwhile, digital media helped us to recall past experiences when we interact with them by registering stories and enabling the retrieval of memories (Erll, 2011). The various forms of life writing and recalling personal pasts were tied to the use of technologies (Ana and Willian, 2019). These technologies also constituted the context of memory production and consumption (Olick, 1999). Digital media opened up new horizons of investigation as they are “fundamentally altering what memory is and what is possible to remember and forget” (Hoskins, 2017). The immediacy, connectivity, and volume of digital data and information enable a participative culture of remembrance (Hoskins and Halstead, 2021). In the context of the pandemic, information about Covid-19 reached individuals through various digital media platforms, enabling them to perceive the occurrence and stage of development of events, then formed corresponding memories. Especially during the lockdown, public online observances sought to commemorate the ongoing Covid-19 crisis, and new forms of remembrance formed a cultural memory as well as a social media memory (Adams and Kopelman, 2021).

### Personal involvement in memory shaping

Personal involvement can be defined as the perceived relevance of an object based on inherent needs (Zaichkowsky, 1985). Individual involvement has been shown to influence memory performance from many perspectives and in many scenarios with its heterogeneous forms across occasions. Television program involvement has previously been shown to enhance advertising memory (Tavassoli et al., 1995). In the field of consumer behavior research, individuals’ different levels of product involvement make their memories of brands vary (Feng et al., 2019). In addition, religious involvement has been shown to predict individuals’ situational memory (Kraal et al., 2019). The level of involvement in video games may explain the memory association with video games (Russell et al., 2020). Moreover, involvement level is also proven to act as a moderator in deception and memory performance (Li and Liu, 2021).

In memory studies, researchers have typically distinguished memories from “first-hand memories,” which are memories of experiences in which people are personally involved rather than having learned about them from an external source (Pillemer, 2009). Particularly, memory performances in disaster events differed depending on the degree of disaster involvement: victims and witnesses remembered more details than controls, and victims remembered central and peripheral details more accurately than other participants (Israel and Irena, 2010). People’s mental state during a disaster also determines how they form flashbulb memories, as involvement in it or non-involvement in it affects their mental state when they recall it, e.g., those with first-hand experience in the fire mentally return to and re-experience the disaster more often and with more intense emotions than those with second-hand experience, which made their flashbulb memories more factual (Knez et al., 2021). Similarly, victims who experienced the Marmara earthquake directly reported higher levels of flashbulb memories than those who only heard it in the news, owing to the higher importance of the earthquake to victims (Er, 2003). Moreover, Americans who were more involved in the 9/11 terrorist attack scored higher on flashbulb memory performances, background knowledge, and emotions than those in other countries who were less involved (Luminet et al., 2004). Researchers also provided evidence from a physiological perspective that participants who were in Downtown Manhattan, close to the World Trade Center, exhibited selective activation of the amygdala as they recalled events from 9/11 while those who were in Midtown did not (Sharot et al., 2007). To summarize, participants’ memories of details related to them at the time of the event differ depending on their involvement (Raw et al., 2021). Flashbulb memories of first-hand experiences are emotionally and cognitively more directive for the self than those of second-hand experiences (Pillemer, 2009).

### Flashbulb memory

Brown and Kulik (1977) first introduced the concept of flashbulb memory (FBM). They administered a questionnaire survey with 80 subjects. Their study tried to investigate whether or not the subjects remembered where they were and what they were doing when they first heard about significant political events such as John Kennedy and Martin Luther King assassinations. They explained, in their article, that the levels of surprise and consequentiality can trigger the formation of Flashbulb Memories (Brown and Kulik, 1977). Flashbulb memory has been suggested as a special category of autobiographical memory, which is a type of personal and collective remembering of emotionally-charged and surprising events (Brown and Kulik, 1977; Pezdek, 2003). The key issue related to FBMs, according to Brown and Kulik, is no memory of the news event itself, but why we remember our personal circumstances for receiving this news (Berntsen, 2009). FBMs are a unique example of memories that are typically formed following a surprising and highly emotional public event such as a national disaster or the death of a well-known public figure (Raw et al., 2021). Unlike other autobiographical memories that contain detailed recollections of the incident at hand, FBM is suggested to be stronger, more enhanced, and more vivid (Bohannon, 1988; Larsen, 1992; Conway et al., 1994). That is, FBMs are usually more accurate and solid than memories of the original event (Curci and Luminet, 2006) and can remain highly persistent and indelible years after the initial event (Brown and Kulik, 1977).

Three properties have been proposed to approximate FBM performance: specificity, confidence, and consistency (Talarico and Rubin, 2003, 2007, 2009; Curci and Luminet, 2006; Lanciano et al., 2013; Curci et al., 2015). FBM specificity is often assessed according to the richness of details in participants’ recollections (Brown and Kulik, 1977; Julian et al., 2009). These details are all about the individual himself/herself at the moment when he/she knew the event: the time, the source of information, the location, the companion, the behavior beforehand, and the behavior after (Brown and Kulik, 1977; Pillemer, 1984; Christianson, 1989; Neisser and Harsch, 1992). FBM confidence is often assessed through self-reported indices. In studies adopting test–retest designs, researchers have usually compared recollections taken at two different points in time to establish the coherence and stability of recalled canonical details to assess FBM consistency (Curci and Luminet, 2006; Luminet and Curci, 2009). Approaches to FBM assessment have been based on explicit self-reported measures such as questionnaires according to individuals’ recalls instead of direct tests (Lanciano et al., 2013). As mentioned above, the procedure of FBM research usually consists of two steps (Brown and Kulik, 1977): test and retest. In the test phase, participants were often asked to report their flashbulb memory properties for a certain event and how confident they were about their recall. Researchers then calculate a total score of FBM specificity and FBM confidence of every participant. In the retest phase, participants in the test phase were invited to report the flashbulb memory characteristics of the event again. After the retest phase, researchers compared the FBM answers of the same participants during two phases to check the consistency of each participant’s flashbulb memory. As for the investigation of antecedent variables of FBM, researchers have examined from different perspectives according to the different features of events, such as different levels of consequentiality caused by the degree of participation in a natural disaster and the severity of the conditions (Neisser et al., 1996; Knez et al., 2021), various FBM performances related to the 9/11 attack event attribute to different nationalities and the regional disparity of America (Hirst et al., 2015; Dégeilh et al., 2021), and individuals’ social identity in events which has specific characteristics like the Pope’s resignation and the UK’s 2016 EU Referendum (Curci et al., 2015; Raw et al., 2021).

### Empathy

In the intersection of social cognition and neuroscience research, clinical researchers argued that empathizing with another person can create hypnosis experiences (Krippner, 2004; Wickramasekera II, 2015). They termed this idea as Empathic Involvement Theory (EIT) and viewed the connotation of empathic involvement as the level of empathy (Wickramasekera II, 2015; Rivers et al., 2016; Vargas, 2016). Empathy frequently has been defined as an emotional reaction to the emotional state or condition of other people (Eisenberg and Fabes, 1998), referring to “a method for putting oneself in another inner life in order to feel, experience, and understand” their world (Suga, 2017). To some extent, it is the ability to understand another’s perspective and to share their feelings (Davis, 1994; Decety and Jackson, 2004; Wondra and Ellsworth, 2015). It is the consequence of self-other merging, providing a bridge between self and other (Decety and Sommerville, 2003). Empathy allows people to cross the boundaries of positionality to more deeply understand the full range of human emotions (Shuman, 2005). In the studies of disaster, empathy is considered both a necessary quality for assistance work and a risk factor for secondary traumatization and compassion fatigue in disaster responders (Figley, 2002). Those who are highly empathic are better at detecting others’ emotions (Olderbak et al., 2014). Besides, empathy is proven to play a mediating role in the good relationship between media depictions of natural disasters and the support they ultimately generate (Oosterhof et al., 2009); Although it has been proven to affect autobiographical memory in both clinical and behavioral domains during memory formation, there are few studies on the correlation between empathy and memory, which is probably due to the reason that in basic psychological research, memory and empathy have traditionally been treated as research topics belonging to different domains of psychology (Wagner et al., 2015). Considering that memory research also has a physiological basis and a social cognitive context and that as an important indicator of emotion (Levine and Pizarro, 2004; Cuff et al., 2016), empathy for victims is spontaneous during the coronavirus pandemic with levels varying from individual to individual, this study was to transfer the concept of empathic involvement as the level of empathy into memory studies, to ascertain how empathic involvement predicts flashbulb memories under the Covid-19 background.

### Digital media dependency

Based on the original Media System Dependency (MSD) theory, perceptions of media messages are dependent on the degree to which audiences are dependent on mass media to satisfy their goals (Ball-Rokeach and DeFleur, 1976). “Dependency may result when an individual ritualistically uses communication channels or instrumentally seeks out certain communication messages” (Rubin and Windahl, 1986). Increased dependence on media to meet individual needs is directly proportional to greater perceived media importance in one’s life and subsequently stronger media effects on one’s attitudes and behaviors (Ball-Rokeach and DeFleur, 1976; Lowrey, 2004). In the modern age, the proliferation of online networks and social media has radically altered the distribution of information (Wen et al., 2020). As media technology continues to advance, high dependency on the Internet and other Internet-based media channels for information become a noticeable phenomenon (Li, 2014). Furthermore, this dependency relationship intensifies during times of uncertainty or crisis such as natural disasters, terrorist attacks, and public health emergencies (Loges, 1994). During the Covid-19 pandemic, under the control measures initiated by governments such as social distancing policies, quarantines, travel restrictions, and lockdowns, people rely on the Internet and social media to buy and sell online, read online, teach and learn online, work from home to carrying on life routines, spontaneously (Ali, 2021).

## Hypotheses development

Memory is always formed within the context of spatial forms. French historians Nora called these spaces” places of memory” (Nora, 1989). In practice, collective memory is inherently dependent on “the interaction between social frame and material space” (Halbwachs and Coser, 1992). As space is critical for memory (Jones, 2011; Fuentealba, 2021), Jones and Garde-Hansen’s research once clarified the connection between the two from the perspective of the geographical attribute of memory: “that of being of past spaces and places as well as past times, and in terms of the prompting and practice of memories by and in current spaces” (Jones and Garde-Hansen, 2012). However, spatial relation in this sense is not only the present relation between the body and the present space but also related to time, containing the self, the past spatial relation, and the memory in the present life (Jones and Garde-Hansen, 2012). Memories of early experiences can evoke a sense of the space in which they took place (Mckinnon et al., 2015). Therefore, in the context of an epidemic event, we take whether the individual was currently in the place where the outbreak occurred (currently in Shanghai in this experiment situation) and whether he had the experience ever being in an epidemic zone during the outbreak of the pandemic in the past as a measure of the individual’s spatiotemporal involvement in the event. Given that those who had first-hand experience at the place and time of the event always had more factual memories (Knez et al., 2021), according to FBM’s three properties as a special form of autobiographical memory, we hypothesize:

*H1*: Spatiotemporal involvement is positively correlated with FBM properties (a) specificity (b) confidence (c) consistency.

It is demonstrated that the underlying mechanisms for empathy and for autobiographical memories were related to a great extent from both psychological and physiological perspectives (Lo, 2021). Through connection with others, one develops a sense of self-superiority about memory (Buckner and Carroll, 2007). In clinical research, empathy has been found to be positively correlated with memory function (Fernandez-Duque et al., 2010). In terms of a specific event, behavioral studies have also shown that when empathy is activated, the psychological mechanisms of autobiographical memory are also triggered (Zaki and Ochsner, 2012), which strengthens the recall process and strengthens the connection between the content of a memory and its source. Empathy strengthens event memory, and empathic arousal prevents overwriting of memory content (Spreng and Grady, 2010). Flashbulb memories, however, tend to focus more on individuals’ circumstances and details when they experienced the event or first heard the news rather than that of the original event (Brown and Kulik, 1977). Given that empathy (Batson et al., 2015), as a moral emotion that forms the basis of altruistic behavior, allows individuals to construct more memories about the fact of the event (Bloise and Johnson, 2007), individuals would pay less attention to their personal details and have a lower level of flashbulb memory. Therefore, we assume that:

*H2*: Empathic involvement is negatively correlated with FBM properties (a) specificity (b) confidence (c) consistency.

As a result of the policy of “lockdown” in the epidemic area, people are increasingly using digital media as a channel for maintaining contact with others during the pandemic (Cornelius et al., 2020). Moreover, benefitting from the accessibility of digital communication devices, individuals are inclined to use digital media to provide informational and emotional support to others (Zhao et al., 2021). In addition, the timing and framing of the information disseminated by media can actually modulate the attention and behaviors of individuals (Funk et al., 2010). Individuals’ media dependency made them scatter digital traces that facilitated a shift in memory from reliance on technology to dependency (Floridi, 2013). Recent research suggests that digital media can facilitate social interactions and offload memory to an external resource (Sharifian and Zahodne, 2020). In this study, digital media dependency is regarded as the degree how much individuals depend on digital media in their daily lives. Given that people with stronger empathy levels are more likely to provide prosocial behaviors (Cuff et al., 2016), during the pandemic, people who are more empathic were more motivated to provide informational and emotional support to others through digital media and rely more on digital media. However, whether digital media dependency would play a role in flashbulb memory formation is uncertain, this study thus hypotheses:

*H3*: Digital media dependency plays a mediating role between spatiotemporal involvement and FBM performance. Specifically, spatiotemporal involvement is positively correlated with digital media dependency, which in turn is positively correlated with FBM properties.

*H4*: Digital media dependency plays a mediating role between empathic involvement and FBM performance. Specifically, empathic involvement is positively correlated with digital media dependency, which in turn is positively correlated with FBM properties.

**Figure 1:**
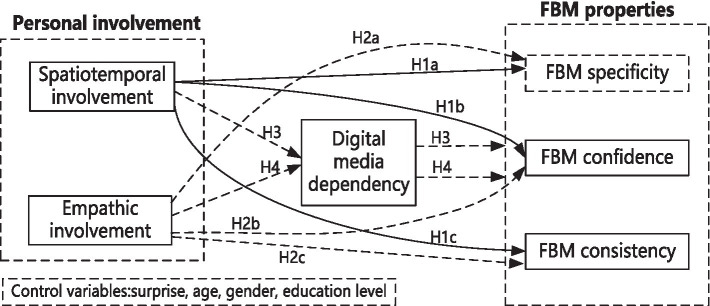
The hypothesized model.

As mentioned above, the hypothesized model of this study is shown in Figure 1, personal involvement is conducted as an independent variable, FBM properties are conducted as dependent variables, and digital media dependency is viewed as a mediation.

## Materials and methods

### Data collection

Methods used for data collection in memory research include written narratives (Shahzad, 2012), diary studies (Yamashiro and Hirst, 2014), in-depth interviews (Adams and Kopelman, 2021), questionnaires (Song and Muschert, 2013), etc. According to the purpose of this study, since the target event was under the background of Covid-19, individuals located where the event broke out were subject to the “lockdown” policy, and the consideration of spatiotemporal involvement required respondents from different locations, this study used an online questionnaire for data collection, which is in line with the traditional self-reported research format used in many FBM studies (Er, 2003; Curci et al., 2015; Raw et al., 2021). The target event selected was the breakout of Covid-19 in Shanghai in late March 2022. Considering that Covid-19 occurred piecemeal in mainland China since the virus appeared, the specialty of the breakout of Covid-19 in Shanghai was even noticeable. As one of the most modern metropolises in China, Shanghai has been widely commended as a model for the precision and efficiency of its Covid-19 prevention and control efforts, and was the first to raise and implement the policy of “dynamic zero.” However, the outbreak of Covid-19 in Shanghai in late March 2022, due to the lack of timely control, the virus spread rapidly and eventually became difficult to control, resulting in a serious chaotic phenomenon of medical resources shortage, lockdown, and quarantine, which shocked the whole China and even the world. During this period, there was numerous online information about Covid-19 in Shanghai which spread out fast, leading individuals’ cognition of the epidemic and the surroundings to change in a day or even a minute. To integrate respondents into the testing environment, before filling in the questionnaire, the respondents were informed of the background of the target event by the instruction part at the head of the questionnaire which says “Since late March, the breakout of Covid-19 in Shanghai has caused public attention. Please finish this questionnaire according to the situation when you first heard about or got to know this incident.” The test phase lasted for 1 week (from May 1, 2022, to May 7, 2022), followed by a one-week retest 10 days later (from May 10, 2022, to May 17, 2022).

The sample of the test phase was originally composed of 553 respondents, mostly aged between 18 and 55. They were given a corresponding bonus after completing the survey. After the data cleaning process (clear invalid questionnaires), 546 respondents were retained (52.2% were females). Ten days later, the respondents of the test phase were invited to take the retest process, and 349 questionnaires were collected. The return rate was 63.9% which was qualified according to the expected return rate between 55 and 70% for each survey in previous longitudinal flashbulb memory studies (Levine and Bluck, 2004; Holland and Kensinger, 2012). The descriptive analysis is shown in Table 1, which indicates the age distribution, gender percentage, and the distribution of education levels of our respondents.

**Table 1:** Descriptive analysis.

Variables	Sample percentage	Sample percentage
	(Test phase)	(Retest phase)
*Age*		
18 and below	1%	0%
19 ~ 25	21.20%	18.90%
26 ~ 35	59.20%	61.60%
36 ~ 45	14.50%	15.50%
46 ~ 55	3.70%	4%
55 and above	0.40%	0%
*Gender*		
Male	47.80%	46.10%
Female	52.20%	53.90%
*Level of education*		
High school and below	12.50%	10.30%
Bachelor’s degree	78.20%	78.80%
Master’s degree	9.10%	10.60%
Doctor’s degree and above	0.20%	0.30%

### Measures

The standard FBM questionnaire used in both the test and retest phases was based on the questionnaire developed by Curci and Luminet and their colleagues (Luminet et al., 2004; Curci and Luminet, 2006). The digital media dependency questionnaire used was based on the media dependency questionnaire developed by Andrew Kennis (Kennis, 2020) and the empathy questionnaire was based on the General Empathy Items developed by Andreychik and Migliaccio (Andreychik and Migliaccio, 2015). FBM specificity, FBM confidence, digital media dependency, and empathy for the Covid-19 pandemic in Shanghai were assessed during the test phase. The retest phase allowed us to assess FBM consistency over time, according to a largely validated procedure (Conway et al., 1994; Curci and Luminet, 2006; Lanciano et al., 2013). During the test phase, respondents individually completed the standard FBM questionnaire and those who had agreed to participate in subsequent phases of data collection were contacted again for the retest phase. Notably, due to the particularity of the location of the epidemic in Shanghai, one of the developed cities in mainland China, the outbreak of the pandemic in Shanghai has caused a big surprise to the public. Given that surprise has been already confirmed as one of the antecedent variables of FBM (Pillemer, 1984; Bohannon, 1988; Conway, 1995; Luminet and Curci, 2009), based on the purpose of this study, the variable surprise was conducted as a control variable. Table 2 shows the measurement and descriptive statistics of the key variables.

**Table 2:** Questionnaire items, means, standard deviations, and reliabilities of the key variables.

Items	Test Phase (Test/retest)	Mean	SD	Cronbach’s α
Spatiotemporal involvement (spatial location and time span)				
(1) Currently in Shanghai				
(2) Currently not in Shanghai but have the epidemic area experience	Test	–	–	–
(3) Currently not in Shanghai and has no epidemic area experience				
Empathic involvement (1 = not at all; 5 = very much)				
(1) You are often “in tune” with other people’s moods				
(2) You always consider other people’s feelings when doing things	Test	3.521	0.597	0.705
(3) You always play the role of “listener”				
(4) Your own emotions are always “following others”				
*FBM specificity*				
(1) The time when you knew the event				
(2) The source of information (e.g., how you knew about the event)				
(3) Your location when you knew the event	Test	8.901	1.49	–
(4) Who you were with when you knew the event				
(5) What you were doing beforehand				
(6) What you did after				
*FBM confidence* (1 = not at all; 5 = very much)				
How much sure you sure about your memory	Test	4.213	0.685	–
*FBM consistency*	Retest	6.373	3.084	–
Digital media dependency				
(1) How much time do you spend using digital media platforms in your daily life (1 = very short; 5 = very long)	Test	3.664	0.745	0.735
(2) How often do you participate in information activities on various digital media platforms in your daily life (1 = hardly; 5 = very often)				
(3) How many kinds of digital media platforms do you use in your daily life (e.g., instant messaging, social media, short videos, q&a, etc.) (1 = none; 5 = diverse)				
(4) How many types of information activities do you conduct on digital media platforms in your daily life (such as sending messages, posting, making comments, likes, etc.) (1 = none; 5 = diverse)				
*Surprise*				
(1) The extent to which the place the pandemic broke out was unexpected for you (1 = not at all; 5 = very much)	Test	3.612	0.891	0.723
(2) The extent to which the status of the pandemic was unexpected for you (1 = not at all; 5 = very much)				

#### Independent variables

Spatiotemporal involvement In the test phase, we invited respondents to report their current location and their epidemic (Covid-19) experience to assess their spatiotemporal involvement. Since the event selected for this study happened in Shanghai, respondents who reported their location currently in Shanghai had the highest level of spatiotemporal involvement, and those whose current location was not in Shanghai but had previous experience in the epidemic area had a medium level of spatiotemporal involvement, and those whose current location was not in Shanghai and had no previous experience in the epidemic area had the lowest level of spatiotemporal involvement.

Empathic involvement as mentioned above, empathic involvement is assessed by the level of empathy. Given that the background information of the target event of the Covid-19 outbreak in Shanghai was instructed at the beginning of the questionnaire as our test situation, it is reasonable to believe the respondents’ answers are attached exactly to the event. During the test phase, items used to assess respondents’ empathic involvement were based on Andreychik and Migliaccio’s General Empathy Items. We made a revision to the original items to match the language context of Chinese according to our experiment background. Four items were finally used: 1) You are often “in tune” with other people’s moods 2) You always consider other people’s feelings when doing things 3) You always play the role of “listener” 4) Your own emotions are always “following others.” Respondents were asked to score the above questions using the Likert scale (1 = not at all; 5 = very much).

#### Dependent variables

FBM specificity At the test phase, respondents need to fill in a questionnaire concerning their reception context of the breakout of the Covid-19 pandemic in Shanghai. This part is aimed to check how vivid and clear their flashbulb memories are with six items corresponding to FBM attributes (Brown and Kulik, 1977; Bohannon, 1988; Finkenauer et al., 1988): (1) the time when they knew the event, (2) the source of information (e.g., how they knew about the event), (3) their location, (4) who they were with, (5) what they were doing beforehand and (6) what they did after. According to the prior study (Curci et al., 2015), to measure FBM specificity, for items (1), and (2), score 1 was assigned if respondents showed an exact recall, score 0 was assigned when the answer was missing or it was incongruent with respect to the question. For items (3), (4), (5), and (6), score 2 was assigned if respondents showed a detailed recall, score 1 was assigned when the answer was partially detailed and score 0 was assigned when the answer was missing or it was incongruent with respect to the question. Scores for each FBM category were summed to get an FBM specificity index.

FBM confidence At the test phase, respondents were asked to evaluate the level of confidence in their recollection, which represents how certain they are about their flashbulb memories of the Covid-19 pandemic in Shanghai, using a Likert scale (1 = not at all; 5 = very much).

FBM consistency After the retest phase, each item answered by the respondents during the retest phase was compared to what they had answered during the test phase in order to check how their FBMs of the two phases are consistent (Curci and Luminet, 2006). During the retest phase, respondents were required to fill in the FBM standard questionnaire again. Accordingly, score 2 was assigned to respondents who performed a completely consistent recall, that is, provided exactly the same answer on both tests (i.e., for the other people’s question: “sister and father” both times). Score 1 was assigned when answers were almost identical but not entirely identical (i.e., “sister and father” at the test and “sister” at the retest). Score 0 was assigned when the answers were either missing in both phases or totally different (i.e., “sister and father” at the test, “boyfriend” or missingthe at retest). Scores for each FBM category were summed up to obtain the FBM consistency index.

#### Mediation variables

Digital media dependency at the test phase, Andrew Kennis’ media Dependency Scale (Kennis, 2020) was used to measure the digital media dependency index of the respondents, which includes four questions. Respondents were asked to score the following questions using Likert scales: 1) How much time do you spend using digital media platforms in your daily life (1 = very short; 5 = very long) 2) How often do you participate in information activities on various digital media platforms in daily life (1 = hardly; 5 = very often) 3) How many kinds of digital media information platforms you use in your daily life (i.e., instant messaging, social media, short videos, online question, and answer, etc.) (1 = none; 5 = diverse) 4) How many types of information activities do you conduct on digital media platforms in your daily life (i.e., send messages, post, make comments, likes, etc.) (1 = none; 5 = diverse).

#### Control variables

Surprise As mentioned above, owing to the special status of Shanghai in mainland China, the outbreak of Covid-19 that occurred in Shanghai has caused surprise to the public. Although surprise has been proven to be one of the factors in FBM performances in classical FBM research (Pillemer, 1984; Rubin and Kozin, 1984; Bohannon, 1988; Conway, 1995; Luminet and Curci, 2009), our research questions did not focus on surprise. Thus, we took it as a control variable in order to eliminate any interference it might have had with the formation of FBMs in our study. Surprise was assessed by two questions. Respondents rated their responses on a 5-point scale (1 = not at all; 5 = very much). The items are: (1) the extent to which the place the pandemic broke out was unexpected for you and (2) the extent to which the status of the pandemic was unexpected for you. Besides, gender, age, and level of education are also taken as control variables to avoid their possible bias in memory formation.

## Results

### Personal involvement on FBM properties

Data from the test phase was used to examine the relationship between spatiotemporal involvement and FBM specificity and FBM confidence. The result of the one-way ANOVA test showed that there were significant differences in FBM specificity performance with different levels of spatiotemporal involvement (*p* < 0.01). Specifically, participants who reported their current location as Shanghai had the highest FBM specificity scores, those who were not currently in Shanghai but had experience in the epidemic area had medium FBM specificity scores, while participants who were not currently in Shanghai and had no experience in the epidemic area had the lowest FBM specificity scores. This indicates that the higher the spatiotemporal involvement degree, the higher the FBM specificity score, that is, the spatiotemporal involvement degree has a significant positive effect on FBM septicity. Thus, hypothesis H1a is supported. However, there is no significant difference between the FBM confidence of participants with different levels of spatiotemporal involvement, and thus hypothesis H1b was not supported. Then, the relationship between spatiotemporal involvement and FBM consistency was tested using the data obtained from the retest phase. The one-way ANOVA test for FBM consistency and spatiotemporal involvement showed that there was a significant difference in FBM consistency performance between levels of spatiotemporal involvement (*p* < 0.01). Participants who reported their current location as in Shanghai had the highest FBM consistency scores, while those who were not currently in Shanghai but had experience in the epidemic area had medium FBM consistency scores, and those who were not currently in Shanghai and had no experience in the epidemic area had the lowest FBM consistency scores. This indicates that the higher the spatiotemporal involvement, the better the FBM consistency, and that spatiotemporal involvement has a significant positive effect on FBM consistency. Thus, hypothesis H1c was supported (see Table 3).

**Table 3:** Spatiotemporal involvement on FBM properties.

Spatiotemporal involvement	FBM specificity (SD)	FBM confidence (SD)	FBM consistency (SD)
Currently in Shanghai	9.0849^***^ (1.417)	4.2703 (0.696)	7.1986^***^ (2.921)
Currently not in Shanghai but have the epidemic area experience	8.9537^***^ (1.300)	4.1666 (0.661)	6^***^ (3.084)
Currently not in Shanghai and have no epidemic area experience	8.0704^***^ (1.922)	4.1408 (0.702)	5.7532^***^ (3.164)
Model	*F* = 13.891	*F* = 1.801 *p* > 0.1	*F* = 9.006 *p* < 0.01

*p < 0.1, **p < 0.05, ***p < 0.01.

In the next step, the data was processed using Stata 15.0 to determine the relationship between empathic involvement and the FBM properties. First, a regression test was made to examine the relationship between FBM specificity, FBM confidence, and empathy, respectively, using the data obtained in the test phase. Then the same process was made on FBM consistency and empathy using data from the retest phase. The variables including surprise, sex, age, education level, and this time also including spatiotemporal involvement are taken as control variables throughout the processes (see Table 4). The results show that empathic involvement has a significant negative effect on FBM specificity, while the effect on both FBM confidence and FBM consistency is not significant. Thus, hypothesis H2a was supported, while hypotheses H2b and H2c were not supported.

**Table 4:** Empathic involvement on FBM properties.

Variables	FBM specificity	FBM confidence	FBM consistency
Empathic involvement	−0.2199^**^	−0.0488	−0.0550
Surprise	0.2537^***^	0.0806^**^	0.3058^*^
Sex	−0.0294^**^	−0.0973	0.1931
Age	0.4639	−0.2085	−0.2722
Education level	−1.0991	−0.7852	−2.2677
Spatiotemporal involvement	0.8766^***^	0.0901	0.9986^***^
Model	*R*^2^ = 0.0459 *F*(12,533) = 2.14 *p* < 0.05	*R*^2^ = 0.0346 *F*(12,533) = 1.59 *p* < 0.05	*R*^2^ = 0.0850 *F*(12,336) = 2.60 *p* < 0.05

*p < 0.1, **p < 0.05, ***p < 0.01.

### Mediation effects of digital media dependency

Based on the above test procedure and results, both spatiotemporal involvement and empathic involvement had significant effects on FBM specificity. This study further examined whether digital media dependency plays a mediating role in the above processes. The mediating effect was examined using Baron and Kenny’s (1986) mediating effect test procedure. First, this study examined the effect of empathic involvement on digital media dependency and conducted a regression analysis of digital media dependency and empathic involvement. The results showed that empathic involvement had a significant positive effect on digital media dependency. Secondly, the study regressed FBM specificity on empathic involvement and digital media dependency at the same time, and the regression coefficients were all significant. It is worth noting that empathic involvement has a significant negative effect on FBM specificity. While empathic involvement has a significant positive effect on digital media dependency, which in turn has a significant positive effect on FBM specificity. This indicates that digital media dependency plays a suppressing effect in the path of empathic involvement affects FBM specificity instead of a mediating effect. To investigate the effect more distinctly, we used the bootstrap test with 1,000 samples at 95% confidence intervals (Preacher and Hayes, 2008) to test the suppressing effect, and the results are displayed in Table 5. Then, this study regressed digital media dependency on spatiotemporal involvement and found that spatiotemporal involvement has no significant effect on digital media dependency. Thus, hypothesis H3 of this study was not supported and hypothesis H4 was partially supported.

**Table 5:** Bootstrapping: Mediation effect test.

Variables	Digital media dependency		FBM specificity	
Empathic involvement	0.2244^***^		−0.2365^**^	
Digital media dependency	–		0.1908^**^	
Age	−0.1361		0.5438	
gender	0.1453		−0.0784	
Education level	0.6074		−1.1056	
Surprise	0.1520^***^		0.1785^**^	
Spatiotemporal involvement	−0.1229		0.8532	
Model	*R*^2^ = 0.1272 *F*(14,531) =5.53 *p* < 0.001		*R*^2^ = 0.0895 *F*(15, 530) = 3.47 *P* < 0.05	
Effect	Boot Effect	Boot SE	Boot LL 95% CI	Boot UL 95% CI
Indirect effect	0.04926	0.0239	0.0025	0.0961
Direct effect	−0.2391	0.1004	−0.4354	−0.0428

*p < 0.1, **p < 0.05, ***p < 0.01.

### Control variables

During the above tests, it can be noticed that as a control variable, surprise has a significant effect on FBM specificity, which again proved that surprise was an important antecedent variable of FBM (Adams and Kopelman, 2021). Especially in the context of this study, the outbreak of Covid-19 occurred in Shanghai, one of the metropolises of China, which caused people to surprise and further deepened people’s impressions and memory.

## Discussions

### FBM performances across different levels of spatiotemporal involvement

The spatiotemporal distance from the event decides whether the individual has first-hand experience of the event, which affects the vividness of the flashbulb memory. Although in some studies, flashbulb memories of public events without close personal experience have in some cases been considered not substantially different from other autobiographical memories (Sharot et al., 2007), they can also serve as witnesses and participate in the meaning-making trauma process (Hirst et al., 2021). In this study, people in almost every area of China were attached to the target event of the outbreak of Covid-19 in Shanghai. People may not be in Shanghai during this outbreak, but the impact of issues such as the subsequent policy and the risk of the epidemic spillover to other provinces and areas are all nationwide. People in China cannot decide whether they are interested in this event or care about it. Everyone has to be a part of this fight and their memories will all be witnesses of this national traumatic event. Based on this background, the current study was to ascertain spatiotemporal involvement and FBM performances of participants upon being notified of the Covid-19 outbreak in Shanghai, it is found that: in terms of FBM specificity, the scores of respondents whose current location is Shanghai are significantly higher than that of respondents who are not currently located in Shanghai but had experience in the epidemic area. While the scores of those whose current location is not in Shanghai but who had experienced in the epidemic area are significantly higher than those who are not located in Shanghai and had no experience in the epidemic area. That is, those with high spatiotemporal involvement perform better in FBM specificity. Similarly, individuals with higher spatiotemporal involvement and higher FBM consistency performed better. The closer in time and space a person is to the event when it occurs, the more accurate and long-lasting (more consistent) the memory of self-relevant details at the time of being informed of the event. In line with the study showing that the public in cities that have been hit by SARS has an earlier, stronger, and more persistent awareness of Covid-19 (Chen et al., 2020), this study reconfirms the impact of spatial distance on memory from the side perspective. In this study, in the context of the outbreak of the pandemic in Shanghai, individuals currently in Shanghai have “personal experience” of the event, and their perception is more “real” and “intense,” “being there” rather than “hearing the news” (Knez et al., 2021). The event may “strike a chord” with people whose current location is not in Shanghai but who had experience in the epidemic area because of the similar experience they have had. They are more inclined to feel the event is a “reproduction” of the “lives” they have experienced elsewhere than people who had no similar experience, which makes their FBMs more specific and deeper. While considering that the test phase was set 1 month after the original breakout and the retest phase was 10 days after the test phase, these performances of FBM consistency may be under a certain time condition. However, in terms of FBM confidence, the effect of spatiotemporal involvement is not significant. The possible reason is that FBM is spontaneous (Abel and Berntsen, 2021; Berntsen, 2021), people do not deliberately remember details related to themselves at the time of being informed other than the original incident, and their confidence in this part of the memory almost has nothing to do with the location at the time of the incident. However, it can be found that participants’ confidence scores were all high (above 4 with a full score of 5) regardless of their level of spatiotemporal involvement, which fits in with previous research: Confidence is often at the ceiling for FBMs and often remains that high for at least months after the event (Christianson, 1989; Christianson and Engelberg, 1999; Niedzwienska, 2003; Weaver III and Krug, 2004; Otani et al., 2005; Talarico and Rubin, 2007). Actually, in some cases, people can have a high degree of confidence in their inaccurate or even false memories. For example, eyewitness testimony is often confabulated, and the correlation between confidence and accuracy on the witness stand is very low (Neisser and Harsch, 1992). Besides, given that confidence was totally assessed as a single question at the end instead of measuring after each detail provided, our results are likely to be measurement-caused. The findings above responded to the rq1 of this study about spatiotemporal involvement’s impact on FBM performances.

### The restraint of empathy on FBM specificity, but not consistency during Covid-19

The information age has encouraged people to acquire information and news reports about Covid-19 events through various digital media and to develop self-awareness and empathy for people and things in the affected areas. Empathic involvement or empathy was found to have a negative effect on FBM specificity, which appears to be inconsistent with previous research on empathy-promoting memory performances (Franca et al., 2014). In the context of this study aiming at flashbulb memory, it turns out to be that the higher the level of empathy is, the weaker the FBM specificity will be. That is, empathy restrains the performances of FBM specificity. In Franca’s research, empathy was positively correlated with the number of memories about the event, while FBM tends to emphasize the details related to oneself when he/she heard of the public event or news (Lanciano et al., 2013), rather than the event itself and details about the event. The present study shows that individuals with a higher degree of empathy were more likely to focus more on the memory of the event itself cognitively, while paying less attention to the details of themselves and their surroundings at the moment they heard of the news, resulting in poorer performance of FBM specificity. In addition, in the context of the “small peak” event of the epidemic, the level of empathy has no significant impact on FBM confidence and FBM consistency, which shows that empathy, as an ability to empathize with others, does not make individuals more or less certain about self-related memories and does not cause self-related memories to change significantly over time, neither. Since FBMs are based on one’s idiosyncratic discovery event, making it impossible to directly assess memory accuracy, and a one-time assessment can be biased by individuals’ confabulation, consistency across testing times has become almost universally regarded as the only acceptable way to best estimate FBM accuracy (Neisser and Harsch, 1992; Talarico and Rubin, 2007; Cordonnier and Luminet, 2021). The initial memory account is assumed to be veridical, so those accounts that share more features across times of testing are also assumed to be the most accurate (Julian et al., 2009). However, some researchers have argued that test–retest consistency cannot logically be equated to accuracy and that two memory accounts can be consistent without being accurate, or accurate without being consistent. Post-event information, like that obtained through recounts, can overwrite a person’s original memory, resulting in the inaccuracy of recall (Loftus et al., 1978). In other fields of memory, there also seems to be no reliable relationship between consistency and accuracy (Smeets et al., 2004). The initial amount of recalling details that refers to the index of FBM specificity may be an alternative way to predict FBM accuracy, and sometimes, better than consistency (Bohannon, 1988; Julian et al., 2009).

However, in the current study, empathic involvement had a negative effect only on specificity but not consistency. People with high empathy levels would pay more attention to the victims or the center of the event and less attention to their surroundings of themselves. This finding was consistent with Pezdek’s (2003) claim that with increasing emotional involvement, autobiographical FBM decreased and event memory increased. Pezdek (2003) developed this theory by finding that participants from Manhattan, presumably most involved in the 9/11 attacks, had the most consistent factual memories and the least detailed autobiographical memories when compared to participants from California and Hawaii. In this study, empathic involvement seems only to help people build a general impression of the moment they heard the news but does not cause these self-related memories to change significantly over time. Surprise had significant positive effects on both specificity and consistency. We speculate that this is because surprise stimulates sustained attention to the event and reminds people of their first encounter with it. Whether people’s memories of the moment they heard about the event are strengthened or forgotten has nothing to do with their empathic involvement, but surprise plays a role. This could also be due to the feature of our target event that the attention of people who initially had a high level of empathy for the victims at the center of the outbreak may be distracted by various corresponding information during the interval between the test and retest, or their memory may be overwritten according to rehearsal or recount through media (Loftus et al., 1978). But surprise of this event towards the place (Shanghai) or the status of the event may last longer which would act as a strengthening effect on memory.

### The suppressing effect of digital media dependency

According to the research hypotheses of this study, based on the significant positive effect of spatiotemporal involvement on FBM specificity and FBM consistency, and the significant negative effect of empathic involvement on FBM specificity, this study further tested whether digital media dependency plays a mediating effect. The findings showed that spatiotemporal involvement had no significant effect on digital media dependency. This indicates that spatiotemporal involvement had a direct impact on FBM specificity and FBM consistency without digital media dependency as a mediation.

Particularly, on the path of empathic involvement affecting FBM specificity, digital media dependency plays a suppressing effect instead of a mediating one. Specifically, although the effect of empathy on FBM specificity is significantly negative, empathy positively affects digital media dependency, and digital media dependency also positively affects FBM specificity. This shows that a deeper degree of empathy leads to a stronger degree of digital media dependency, which will weaken the negative impact of empathy on FBM specificity. Digital media relies on its technical characteristics to strengthen people’s memory by giving them a sense of participation in the memory of Covid-19 (Hoskins and Halstead, 2021). Besides, in the event of a pandemic disaster, individuals with higher levels of empathy are more likely to offer emotional and information support through digital devices by tracking the progress of the disaster and the living conditions of the victims (Qin et al., 2022), increasing their level of reliance on digital media. The information they encountered on digital media about the ongoing event may consistently evoke their memory of the moment they heard of the news, making their FBM specificity perform better, which means that reliance on digital media will help individuals with higher empathy levels to remember more details about themselves at the moment they heard of the event. To the extent that encounters with relevant information through digital media reproduce the event for people, it can be seen as a new form of rehearsal that includes both overt and covert aspects in the digital age. In this regard, our results are consistent with previous studies that frequent rehearsal makes elaborate FBM accounts more accessible (Luminet et al., 2004) or even directly determines FBM (Tinti et al., 2014). Especially, overt rehearsal is more than a simple reproduction of the events constituting the memory. It improves individuals’ FBM by consolidating existing memory traces, that is, the memory for the reception context is improved by being exposed to media (Finkenauer et al., 1988; Pillemer, 2003).

### Theoretical and practical implications

Consistent with the previous studies that those with first-hand experience mentally return to and re-experience more often and with more intense emotions than those with second-hand experience, which made their flashbulb memories more factual (Knez et al., 2021), this study demonstrated that people who are closer in time and space to the outbreak had greater FBM performances under the situation of the “small peak” event of Covid-19. Surprise, as an important determinant of FBM according to classic FBM literature, is also confirmed again in the current study to positively affect FBM (Pillemer, 1984; Bohannon, 1988; Conway, 1995; Luminet and Curci, 2009). Under the peculiar situation of preventing Covid-19 in mainland China now, it relates to everyone, but to varying degrees. This study leads to a new perspective on FBM by transferring the concept of empathic involvement. However, which seems inconsistent with the previous FBM findings is that empathic involvement was negatively correlated with FBM specificity, compared to those who view personal involvement as some other forms such as social/national membership (Kvavilashvili et al., 2003; Curci and Luminet, 2006; Tinti et al., 2009), political stance (Raw et al., 2021), and religious faith (Tinti et al., 2009; Curci et al., 2015). Consistency in our study was less correlated with empathic involvement, which may seem to be different from the propositions that individuals are likely to maintain a consistent memory for these events which are highly consequential for themselves (Luminet et al., 2004; Curci et al., 2015). However, the certain difference between empathic involvement and consequentiality is that the former is more of the ability to share others’ feelings but not the importance to the individuals themselves. Besides, this study again confirmed that confidence can be strong even if the memory is inaccurate (Christianson, 1989; Christianson and Engelberg, 1999; Niedzwienska, 2003; Weaver III and Krug, 2004; Otani et al., 2005; Talarico and Rubin, 2007).

The findings of this study indicated the comprehensive effects of personal involvement on flashbulb memory, carrying several theoretical implications as follows. First, based on the nonnegligible effect of personal involvement on flashbulb memory (Neisser et al., 1996), and the heterogeneousness of personal involvement in different situations (Curci et al., 2015; Hirst and Echterhoff, 2018; Raw et al., 2021), this study explored the concept of personal involvement in a more general way including two aspects: spatiotemporal involvement and empathic involvement for the first time, explaining the connotation of personal involvement from a more general perspective for the epidemic, a catastrophic event common to all mankind, which enriches FBM research. With the advent of digital media, it is very convenient for people to get information and news about events that are not their own experiences, generating cognition and emotion for people and things in disaster areas. Based on the empirical evidence, we demonstrated that empathy negatively affected flashbulb memory performance in this study. Finally, we innovatively incorporate digital media dependency into FBM studies and examine the role of digital media dependency between empathy and FBM specificity. At a time when digital media has become the main channel for information acquisition, it is vulnerable to becoming dependent on it. And less attention has been paid to its effect on memory. It is found that although empathy had a negative effect on FBM, this negative effect was attenuated by digital media dependency, which played a suppressing effect on the path of empathy affecting FBM specificity.

The findings of this study provide important practical implications for the collective memory of the pandemic and the information dissemination and digital media design during the “small peak” events of the pandemic. As an extraordinary moment in human history, people are trying to remember this pandemic in many ways (Historic England, 2020). FBM, as an important part of the autobiographical memory with individualized and personal characteristics, plays an important role in constructing the collective memory of the pandemic. In a situation where the pandemic is not over yet, and the “small peak” events continue to break out, the performance of FBM is different when the spatiotemporal and empathic involvement of individuals are on different levels. However, the epidemic occurred in an era dominated by digital media (Poell, 2020) when people can use digital media to construct memories of the epidemic. According to the results of this study, digital media dependency can weaken the negative impact of empathy on FBM specificity. For individuals with stronger empathy levels, promoting their use of digital media has an enhanced effect on FBM specificity, so digital media or institutions can increase the use of digital media for those who have higher empathy levels by enriching epidemic information on digital media or improving digital media design to facilitate the building of their personalized memory of the pandemic.

## Conclusion, limitations, and future research

### Conclusion

This study investigated the impact of personal involvement on flashbulb memory (FBM) performance in the context of the “small peak” event of the Covid-19 epidemic by conducting a two-stage online questionnaire collection, including a test phase and a retest phase, with special consideration of the role played by digital media dependency. This study measured personal involvement from two aspects: spatiotemporal involvement and empathic involvement, and separately considered their impact on FBM performances. The results show that spatiotemporal involvement has a significant positive impact on FBM specificity and FBM consistency, while empathic involvement has a significant negative impact on FBM specificity. In particular, digital media dependency exerts a surprising effect on empathic involvement and FBM specificity. The findings of this study enrich the research on disaster memory and digital media dissemination, providing not only guidance for the post-disaster recovery process of the pandemic but also a social reference for social media information dissemination and the construction of collective memory.

### Limitations and future research

First of all, the data collection method of this study is only conducted through online questionnaires. Compared with in-depth interviews and offline questionnaires, it is more difficult to observe the status of the respondents while filling in the questions, and it is hard to ensure whether the respondents fill in carefully or understand the meaning of each item sufficiently. A variety of research methods can be used in future research to ensure that the subjects understand the background of the event and the meaning of the item, so as to obtain more vivid memory experience responses. Secondly, the research objectives of this study are only for those who can use digital media expertly. For individuals who are not proficient in using digital media, such as the elderly, children, etc., FBM about Covid-19 events has not been considered. Given that this study examines the use of digital media from a general perspective, the categories of digital media also lack classification. In the future, FBM research on events related to the pandemic can be carried out for groups who are not proficient in using digital media. The effect of different digital media categories should also be taken into consideration. Finally, the research scene of this study is only in mainland China. However, given that Covid-19 is a global issue, the research model of this study can be modified for different countries or ethnic groups and also take into account the other possible factors such as control of governments over media and individuals’ tiredness of “lockdown” and closure in the future to enhance the robustness of the conclusions.

## Data availability statement

The raw data supporting the conclusions of this article will be made available by the authors, without undue reservation.

## Ethics statement

The studies involving human participants were reviewed and approved by School of Journalism and New Media, Xi’an Jiaotong University, China. The patients/participants provided their written informed consent to participate in this study.

## Author contributions

XM and JW contributed to the conception and design of the study. JW performed the statistical analysis and wrote the first draft of the manuscript. XM contributed to the manuscript revision, and read, and approved the submitted version. All authors contributed to the article and approved the submitted version.

## Funding

This work was supported by the National Natural Science Foundation of China (72174164), the National Social Science Foundation of China (Major program) (21&ZD320), and the Social Science Foundation of Shaanxi Province of China (2021M004).

## Conflict of interest

The authors declare that the research was conducted in the absence of any commercial or financial relationships that could be construed as a potential conflict of interest.

## Publisher’s note

All claims expressed in this article are solely those of the authors and do not necessarily represent those of their affiliated organizations, or those of the publisher, the editors and the reviewers. Any product that may be evaluated in this article, or claim that may be made by its manufacturer, is not guaranteed or endorsed by the publisher.
